# Nutrient Intake Values for Folate during Pregnancy and Lactation Vary Widely around the World

**DOI:** 10.3390/nu5103920

**Published:** 2013-09-30

**Authors:** Rosemary A. Stamm, Lisa A. Houghton

**Affiliations:** Department of Human Nutrition, University of Otago, Dunedin 9016, New Zealand; E-Mail: rosemary.stamm@otago.ac.nz

**Keywords:** folate requirements, Nutrient Intake Values, pregnancy, lactation

## Abstract

Folate is a B-vitamin with particular importance during reproduction due to its role in the synthesis and maintenance of DNA. Folate is well known for its role in preventing neural tube defects (NTDs) during the periconceptional period. There is also an increased need for folate throughout pregnancy to support optimal growth and development of the fetus and blood volume expansion and tissue growth of the mother. During lactation, women are at risk of folate deficiency due to increased demands to accommodate milk folate levels. Nutrient Intake Values (NIVs) for folate have been calculated to take into account additional needs during pregnancy and lactation. However, these values vary widely between countries. For example, the folate requirement that is set to meet the needs of almost all healthy women during pregnancy varies from 300 µg/day in the United Kingdom to 750 µg/day in Mexico. Currently, there is no accepted standardized terminology or framework for establishing NIVs. This article reviews country-specific NIVs for folate during pregnancy and lactation and the basis for setting these reference values.

## 1. Introduction

Folate is a B-vitamin involved in one-carbon transfer reactions and plays a fundamental role in nucleotide biosynthesis and methylation reactions [1]. During pregnancy and lactation, the demand for folate is increased to support both the normal physiological changes of the mother and optimal growth and development of the fetus and offspring [2,3]. Observational studies in the 1960s demonstrated higher rates of folate-related anemia (megaloblastic anemia) in unsupplemented pregnant and lactating mothers [4,5,6]. As a result, a number of countries recommended supplementation of folic acid during the prenatal period [7]. Years later, one of the better-known consequences of low folate status emerged with scientific evidence supporting the use of periconceptional folic acid supplementation for the prevention of neural tube defects (NTDs) [8]. While other pregnancy complications have been associated with folate deficiency, data are limited and the findings unclear. More recently, studies have suggested improved neurodevelopment outcomes in children of mothers with higher blood folate concentrations or mothers receiving prenatal folic acid supplements [9,10,11,12]. Thus, establishing nutrient intake recommendations to optimize folate status for pregnant women remains challenging. Moreover, the dietary contributors to folate intake have evolved since the first nutrient recommendations for folate, with an increasing number of countries mandating folic acid fortification of their food supply [8]. Limitations in our understanding of folate bioavailability make accurate interpretation of dietary intakes difficult.

Despite the challenges, most countries have established recommended folate intakes for their populations; however, requirements among countries vary considerably due to a lack of a standardized framework for deriving these standards. In 2005, the United Nations University’s Food and Nutrition Programme, in collaboration with the Food and Agriculture Organization (FAO), the World Health Organization (WHO) and UNICEF, assembled an international working group to establish a common approach for establishing nutrient intake recommendations. The umbrella term Nutrient Intake Values (NIVs) was proposed to refer to a set of specific nutrient standards and is synonymous with the U.S. and Canada Dietary Reference Intakes (DRIs) and the U.K. Dietary Reference Values (DRVs) among others [13,14].

In this paper, we will briefly review the approach used for deriving folate requirements, including considerations of the indicators used to establish adequate intakes, folate bioavailability and the potential influence of gene polymorphisms on requirements. The next section will compare current folate reference values from over 50 countries and describe the scientific foundation of the NIVs for non-pregnant, non-lactating (NPNL), pregnant, and lactating women.

## 2. Terminology

The terms Average Nutrient Requirement (ANR), Individual Nutrient Level (INL_x_) and the Upper Nutrient Level (UNL) will be used throughout the article as they are aligned with proposed common terminology of the international working group [14]. Table 1 provides a list of comparable terminology currently used by different countries and organizations. The ANR is synonymous with the U.S. and the U.K. Estimated Average Intake (EAR) or the European Community Average Requirement (AR). The ANR is derived from a statistical distribution of requirements for a criterion, and is the value that should be used for assessing the adequacy of population intakes and planning of group diets [15]. The ANR also serves as a basis for determining the INL_x_—the recommended intake level for all healthy individuals in the population. An INL_x_ set at 2 standard deviations above the ANR would be INL_98_, covering 98% of the population nutrient requirement. This value is equivalent to the U.S. Recommended Dietary Allowance (RDA), and U.K. Recommended Nutrient Intake (RNI) and the European Community Population Reference Intake (PRI). The INL_x_ should not be used for assessing the adequacy of population intakes; rather it should be used for planning individual’s intakes [15].

**Table 1:** Sources of country Nutrient Intake Values and country specific terminology used for folate Nutrient Intake Values.

Country	Year	Equivalent Term Used for NIVs for Folate				Adapted from	Reference
		NIV	ANR	INL_98_	SI		
Australia and New Zealand	2005	NRV	EAR	RDI	−	U.S.	[16]
Austria, Germany, and Switzerland	2000	†	−	†	−	Own (DACH)	[17]
Belgium	2009	−	−	PRI	−	EC	[18]
Brazil	2000	−	−	IDR	−	WHO	[19]
Bulgaria	2005	−	−	†	−	U.S.	[20]
Central America ^1^ and Panama	1994	−	−	RDD	−	WHO/FAO 1988	[21]
China	2010	DRI	EAR	RNI	−	U.S.	[22]
Colombia	1988	−	−	†	−	Own	[23]
Croatia	2004	−	−	†	−	EC and U.S.	[24]
Cuba	2008	−	−	DRI	−	U.S./WHO	[25]
Denmark, Iceland and Sweden	2004	NNR	AR	RI	−	Own (NNR)	[26]
Estonia	2006	−	−	†	−	NNR	[27]
European Community	1993	RDA	AR	PRI	−	Own (EC)	[28]
Finland	2005	NNR	AR	RI	−	NNR	[29]
France	2001	−	−	−	ANC	Own	[30]
Greece	1993	RDA	AR	PRI	−	EC	[31]
Hungary	2005	−	−	−	†	EC and U.S.	[32]
India	2010	−	−	RDA	−	Own	[33]
Ireland	1999	−	−	RDA	−	Own	[34]
Italy	2012	LARN	AR	PRI	−	U.S.	[35]
Japan	2005	DRI	EAR	RDA	−	Own	[36]
Latvia	2008	−	−	†	−	Own	[37]
Lithuania	1999	RPN	−	RP	−	Own	[38]
Mexico	2004	VNR	RNP	−	IDS	U.S.	[39]
Norway	2005	NNR	AR	RI	−	NNR	[40]
Poland	2008	−	EAR	RDA	−	U.S.	[41]
Republic of Macedonia	2001	−	−	†	−	EC	[42]
Republica Srpska, Bosnia and Herzegovina	2004	−	−	†	−	WHO/FAO	[43]
Russian Federation	2008	−	−	†	−	U.S.	[44]
Slovakia	1997	−	−	OVD	−	Own and WHO/FAO	[45]
Slovenia	2004	−	−	PVD	−	DACH	[46]
South Korea	2010	KDRI	EAR	RI	−	U.S.	[47]
Southeast Asia Region ^2^	2005	−	−	RDA	−	U.S.	[48]
Spain	2010	−	−	IDR	−	Own and Ireland	[49]
The Netherlands	2003	DRI	EAR	RDA ^3^	AI ^4^	Own	[50]
United Kingdom	1991	DRV	EAR	RNI	−	Own	[51]
United States and Canada	1998	DRI	EAR	RDA	−	Own (U.S.)	[52]
WHO/FAO	2004	−	EAR	RNI	−	U.S.	[53]

AI: adequate intake; ANC: apports nutritionnels conseillés; ANR: average nutrient requirement; AR: average requirement; DACH: Germany, Austria, and Switzerland; DRI: dietary reference intake; DRV: dietary reference value; EAR: estimated average requirement; EC: European community; FAO: Food and Agriculture Organization; IDR: ingestión diaria recomendada (Brazil); IDR: ingestas dietéticas de referencia (Spain); IDS: ingestión diaria sugerida; INL: individual nutrient level; KRDI: Korean Dietary Reference Intakes; LARN: livelli di assunzione di riferimento di nutrienti; NIV: nutrient intake value; NNR: Nordic nutrition recommendations; NRV: nutrient reference values; OVD: odporúčané výživové dávky; PRI: population reference intake; PVD: priporočeni dnevni vnosi; RI: recommended intake; RDA: recommended dietary allowance; RDD: recomendiaciones dietéticas diarias; RDI: recommended dietary intake; RNI: recommended nutrient intake; RNP: requerimiento nutrimental promedio; RP: rekomenduotinus paros poreikias; RPN: rekomenduojamos paros maistinių medžiagų ir energijos normos; SI: safe intake; VNR: valores nutrimentales de referencia; WHO: World Health Organization; † Equivalent terms are used; ^1^ inclusive of Belize, Costa Rica, El Salvador, Guatemala, Honduras, Nicaragua, and the Dominican Republic; ^2^ inclusive of Cambodia, Indonesia, Laos, Malaysia, Myanmar, Philippines, Singapore, Thailand, and Vietnam; ^3^ for NPNL women; ^4^ for pregnant and lactating women.

## 3. Establishing Nutrient Intake Values for Folate

### 3.1. Selection of Model Indicators for Folate Adequacy

Selecting a reliable indicator for nutrient adequacy is a critical step in determining the ANR for a subpopulation. Ideally the indicator chosen should be reflective of a physiologic state representative of adequate intake in all individuals; resistant to short-term dietary intake changes and other environmental influences such as infection; easily measurable and non-invasive; elicit a dose-response relationship across a range of intakes; and be accepted worldwide to allow for harmonization of NIVs [13].

Folate requirements in most countries have been established based on concentrations of serum or erythrocyte folate known to be associated megaloblastic anemia—the clinical manifestation of folate deficiency. Several countries, namely Austria, Germany and Switzerland (abbreviated as the DACH countries) and France have chosen concentration of homocysteine (which increases when folate status is low) as an indicator of adequate intake on the basis of its association with CVD risk [17,30]. Although homocysteine concentration is considered a sensitive indicator, it is not specific for folate since it is influenced by nutritional status of other B-vitamins, one-carbon metabolism related nutrients and renal insufficiency [54]. Furthermore, a recent meta-analysis of randomized controlled trials found no significant effect of lowering homocysteine levels with folic acid supplementation on cardiovascular events [55]. In contrast, erythrocyte folate concentration appropriately fits the criteria as an indicator of adequacy. Erythrocyte folate concentration is a marker of long-term folate status (*i.e.*, resistant to recent or transient changes in dietary folate intake), reflects tissue folate stores [56], and shows a dose-response relationship with intake. Erythrocyte folate cut-offs as an indicator of adequacy vary from country to country, ranging from 300 nmol/L to 327 nmol/L [26,28,50,51,52]. Conventionally, folate deficiency has been defined as a serum folate concentration <3 ng/mL (6.8 nmol/L) and an erythrocyte folate concentration <140 ng/mL (317 nmol/L), below which macrocytic anemia is most likely to appear (Folate cut-offs are originally reported in units of ng/mL. To obtain units in nmol/L values have been multiplied by 2.265). These cut-offs were based on data generated from folate depletion studies conducted in a small number of subjects [57,58,59], and erythrocyte folate concentration levels obtained from patients presenting with megaloblastic anemia [60,61]. In addition, the risk of uracil misincorporation into DNA and chromosomal breakage has shown to increase in individuals with erythrocyte folate concentrations below 317 nmol/L [62].

A major difficulty in establishing a scientific consensus for folate deficiency lies in the existence of a number of methodological issues related to the measurement of blood folate. The microbiologic assay is the method of choice for many research laboratories, however, changes in the assay protocol over time have resulted in substantial inter-laboratory differences in the quantification of folate, presenting challenges in data interpretation across studies [63,64,65]. Standardization of folate measurement is needed for accurate assessment of blood folate status. Recent development of sophisticated mass spectrometric techniques have led to the development of serum folate reference measurement procedures [66,67,68]; however, similar procedures for erythrocyte blood folate are still in development [69,70,71]. Once established, there will be a need for clinical trials that relate accurately measured folate biomarker data to clinical outcomes.

### 3.2. Consideration of Folate Bioavailability

Bioavailability is an important factor to consider when estimating folate requirements due to differences in the absorption of naturally occurring food folate and the more bioavailable synthetic folic acid used in supplements and fortified foods. A number of studies used to determine NIVs have been based on data obtained from folic acid supplement trials, causing uncertainties in determining the equivalent amount of the less bioavailable food folate required to maintain adequate folate status. The U.S. DRI process has recommended the use of dietary folate equivalents (DFEs) for planning and evaluating the adequacy of folate intake. DFEs are defined as the amount of naturally occurring food folate plus 1.7 times the amount of folic acid from fortified foods [52]. Several countries, including a number of European countries, India, South Korea, China, Australia and New Zealand have also expressed NIVs in units of DFEs (Table 1). While adjusting for differences in folate bioavailability is particularly important in countries where folic acid has been added to the food supply, the bioavailability estimate for food folate derived from the DFE equation remains questionable. A thorough review of folate bioavailability is presented elsewhere in this special issue [72]. In summary, there are fairly large discrepancies in the bioavailability of naturally occurring food folate with values ranging from 30% [73] to 98% [74] relative to folic acid. These differences are likely attributed to the use of different test foods, difficulties in quantifying the amount of folate in these foods, and metabolic differences in the physiologic response between naturally occurring food folate and the synthetic, fully oxidized, monoglutamate form of folic acid. It should also be noted that a number of trials have shown that [6S]-5-methyltetrahydrofolate in its monoglutamate form given as a supplement or microencapsulated in food results in a similar change in blood folate concentration to folic acid [75,76,77]. Further investigations are required to derive reasonably accurate and precise estimates of folate bioavailability. Recent efforts to scale up folic acid fortification in both developed and developing countries indicate that this should be a research priority.

### 3.3. Genetic Variation in Requirements

Recent advances in the field of genetics and nutrition have highlighted the importance of gene-diet interactions, and introduced the concept of applying genomic knowledge to population-based dietary recommendations. Currently NIVs for folate are targeted to the general and supposedly “normal” population. However, the identification of several genetic polymorphisms in folate metabolism has stimulated research on the impact of these variants on population health [78].

The *C677T* polymorphism in the enzyme methylenetetrahydrofolate reductase (MTHFR) is one of the most investigated genes in folate metabolism. For *TT* homozygous individuals, the polymorphism results in partial enzyme deficiency [78]. The distribution of the *T* allele varies substantially among ethnic groups, with a lower prevalence of the *TT* genotype among Sub-Saharan Africans (0%–2%), North American whites (8%–14%) and Northern Europeans (6%–14%) compared to Southern Europeans (15%–24%) and Hispanic populations (15%–35%) [78,79,80]. The *TT* genotype is associated with lower folate status by 10%–35% and elevated homocysteine concentrations [78]. Consequently folate requirements may be higher in persons with the *TT* genotype, although differences in indicators of folate status among genotype groups appear to be greater when folate status is low [78,81,82,83]. Moreover, data on variability and the magnitude of the effect of the *TT* genotype on requirements are lacking. Nonetheless, Mexico have set requirements based on the U.S. DRIs with an adjustment toward higher intake levels due to the increased frequency of the *TT* genotype in the Mexican population [39].

With the rapid pace of development in genome-wide technology, an increasing number of common genetic variants in folate metabolism will be identified and future research will continue to explore their link with common disorders. As a result, both the genetic and non-genetic variation in requirements within a population will need to be carefully considered in the future development of NIVs. Current evidence suggests that the allowance for variability used in setting INL_98_ levels may already act as a built-in safeguard for the presence of genetic variability within populations [84]. For example, Robitaille *et al*. [85] modeled the effect of *MTHFR TT* prevalence on the IOM RDA demonstrating that even when accounting for a large effect size of 50% (*i.e.*, 50% higher requirement of dietary folate for individuals with a *TT* genotype), the RDA would only increase from 400 µg DFE to 436 µg DFE in a population with 20.3% *TT* genotyped individuals.

### 3.4. Estimating Variability in Requirements

Folate needs vary between individuals, yet information from which to obtain a reliable estimate of the variance or standard deviation of the ANR is limited. When the variance is not known for a nutrient, then a symmetrical distribution is assumed and a coefficient of variation (CV) can be applied—often this is equal to about 10%. For folate, CVs vary widely; 10% has been used in Japan, the U.S. and Canada, 15% in the DACH countries and the U.K., 20% in the European Community and Mexico, and 25% in the Netherlands and the Nordic countries (Denmark, Finland, Iceland, Norway and Sweden). Given the INL_x_ is derived from the ANR and its distribution, these differences in the CV assumed has lead to some of the discrepancy in the INL_98_ set among countries. For example, both Japan and the Netherlands have set the same ANR value of 200 µg DFE, however Japan have assigned a CV of 10% [36], while the Netherlands have assigned a CV of 25% [50] leading to a discrepancy of 60 µg DFE in their INL_98_. In the face of limited data, the uncertainty surrounding the variation in requirements leads to the potential to over-estimate the proportion of inadequate intakes if the CV is set too low compared to the actual variability in requirements of a population, or under-estimate the proportion of inadequate intakes if set too high.

The traditional approach to determining nutrient requirements is to test a range of nutrient intakes (preferably from foods) in the same individuals using a controlled diet over a sufficient duration to elicit a response in the nutritional indices measured. Often participant numbers in these types of controlled metabolic-unit depletion/repletion and nutrient balance studies are small and consequently, inter-individual variation in requirements cannot be determined. Although challenging, rigorous large-scale controlled-diet studies on free-living subjects are needed to allow for a reasonable approximation of the variability in requirements and the distribution of intakes.

## 4. Nutrient Intake Values for Folate in Use around the World

NIVs for folate for NPNL, pregnant and lactating women by country are listed in Table 2. While several countries have established their own NIVs for folate, many others including the WHO have either shared or adopted values from other countries (Table 1). The most commonly adopted NIVs are those set by the IOM in 1998. Although folate NIVs for pregnant and lactating women are consistently higher than for NPNL women, NIVs vary among countries and the use of DFE units is inconsistent. Moreover, many countries have only specified a value equivalent to the INL_98_.

**Table 2:** Nutrient intake values for non-pregnant, non-lactating (NPNL), pregnant, and lactating women by country.

Country	Units	NPNL		Pregnant		Lactating	
		ANR	INL_98_	ANR	INL_98_	ANR	INL_98_
Australia and New Zealand ^1^	µg DFE	320	400	520	600	450	500
Austria, Germany, and Switzerland ^1^	µg DFE	−	400	−	600	−	600
Austria, Germany, and Switzerland ^1,2^[86]	µg DFE	220	300	420	550	340	450
Belgium	µg	−	200	−	400	−	350
Brazil	µg	−	240	−	355	−	295
Bulgaria	µg DFE	−	400	−	600	−	500
Central America ^3^ and Panama	µg	−	170	−	370–470	−	270
China	µg DFE	320	400	520	600	450	500
Colombia	µg	−	160	−	460	−	360
Croatia	µg DFE	−	200	−	600	−	500
Cuba	µg DFE	−	400	−	600	−	500
Denmark, Iceland and Sweden ^1^	µg	200	400 ^5^	−	500	−	500
Estonia	µg	−	400 ^5^	−	500	−	500
European Community	µg	140	200	−	400	−	350
Finland ^1^	µg	200	400 ^5^	−	400	−	400
France ^1^	µg	−	300 ^6^	−	400 ^6^	−	400 ^6^
Greece	µg	140	200	−	400	−	350
Hungary ^1^	µg	−	200 ^6^, 400 ^6,7^	−	400 ^6^, 600 ^6,7^	−	350 ^6^, 500 ^6,7^
India	µg DFE	−	200	−	500	−	300
Ireland ^1^	µg	−	300	−	500	−	400
Italy ^1^	µg DFE	320	400	520	600	450	500
Japan	µg	200	240	370	440	280	340
Latvia	µg	−	300	−	400	−	300
Lithuania	µg	−	300	−	400	−	480
Mexico	µg DFE	320	460 ^6^	520	750 ^6^	450	650 ^6^
Norway ^1^	µg	200	400 ^4^	−	400	−	400
Poland ^1^	µg DFE	320	400	520	600	450	500
Republic of Macedonia	µg	−	200	−	400	−	500
Republica Srpska, Bosnia and Herzegovina	µg DFE	−	400	−	600	−	500
Russian Federation	µg	−	400	−	600	−	500
Slovakia	µg	−	200	−	400	−	300
Slovenia	µg DFE	−	400	−	600	−	600
South Korea	µg DFE	320	400	520	600	450	550
Southeast Asia Region ^4^	µg	−	400	−	600	−	500
Spain ^1^	µg	−	300	−	500	−	400
The Netherlands ^1^	µg DFE	200	300	−	400 ^6^	−	400 ^6^
United Kingdom ^1^	µg	150	200	−	300	−	260
United States and Canada ^1^	µg DFE	320	400	520	600	450	500
United States 1989 [87]	µg	−	180	−	400	−	280 ^8^, 260 ^9^
United States 1980 [88]	µg	−	400	−	800	−	500
United States 1974 [89]	µg	−	400	−	800	−	600
United States 1968 [90]	µg	−	400	−	800	−	500
WHO/FAO	µg DFE	320	400	520	600	450	500
WHO/FAO 1988 [91]	µg	−	170 ^6^	−	370–400 ^6^	−	270 ^6^

NR: average nutrient requirement; DFE: dietary folate equivalents; FAO: Food and Agriculture Organization; INL: individual nutrient level; NPNL: non-pregnant, non-lactation; WHO: World Health Organization; ^1^ These countries recommend an additional intake of 400 µg folic acid per day from supplements and/or fortified foods for women who are capable of becoming pregnant or planning a pregnancy, or who have an unbalanced poor diet, or because women are unlikely to meet the NIV, for the prevention of neural tube defects. Poland does not specify a dosage for folic acid supplementation [16,34,50,52,86,92,93]. ^2^ Proposed values to be approved August 2013; ^3^ Inclusive of Belize, Costa Rica, El Salvador, Guatemala, Honduras, Nicaragua, and the Dominican Republic; ^4^ Inclusive of Cambodia, Indonesia, Laos, Malaysia, Myanmar, Philippines, Singapore, Thailand, and Vietnam; ^5^ The Nordic recommendations presented are for women of reproductive potential; ^6^ the equivalent of a safe intake level is used rather than an INL_98_; ^7^ INL_98_ when cereals are supplemented; ^8^ 1st 6 months; ^9^ 2nd 6 months.

### 4.1. Non-Pregnant, Non-Lactating (NPNL) Women

The INL_98_ for NPNL women varies between 200 µg and 460 µg DFE per day. Early work by Herbert in 1962 in one adult male and three adult females established that a minimum of 50 µg folic acid per day was required to recover normal hematology after a prolonged folate deficient diet [58,94]. The European Community have used this data to establish ANR for folate of 140 µg/day with the reasoning that the ANR would be somewhat higher than the minimum 50 µg folic acid/day—setting the value at 70 µg folic acid/day multiplied by a bioavailability correction factor of 2. Later work published by Sauberlich *et al.* in 1987 [95] and O’Keefe *et al.* in 1995 [96] generated from controlled metabolic studies, suggested higher intake levels of between 200 µg and 320 µg dietary folate/day were required to maintain status and prevent deficiency in NPNL women. The IOM (U.S. and Canada) and the Netherlands have considered much of the same data in determining NIVs for NPNL women, including the studies of Sauberlich *et al.* [95] and O’Keefe *et al.* [96] together with studies of Milne *et al.* [97] and Jacob *et al.* [98]. The IOM chose an ANR of 320 µg DFE/day, based primarily on data from the study of O’Keefe *et al.* [96], which demonstrated that at this intake level, 3 out of 5 women had an erythrocyte folate concentration below 305 nmol/L and serum folate below 7 nmol/L, and 2 out of 5 women had a homocysteine concentration greater than 16 nmol/L with another participant above 14 nmol/L. These data suggested that approximately half of women would have a normal erythrocyte folate level at an intake of 320 µg DFE/day [52]. In contrast, the Netherlands chose a lower ANR of 200 µg DFE/day, relying primarily on data from the depletion-repletion study of Sauberlich *et al.* [95], in which 200 µg of folate from food sources resulted in stabilization of plasma folate concentration in 2 out of 3 subjects [50,95]. Data from the study of Milne *et al.* [97] of adult men was also used to support the Netherland’s ANR of 200 µg DFE/day. Finally, the NIVs for folate set by the DACH countries are currently based on data from O’Keefe *et al.* [96], but are in the process of being revised toward lower NIVs on the basis of data from the studies of Sauberlich *et al.* [95] and Milne *et al.* [86,97].

The small study sample sizes of between 3 and 6 participants per test group, and the uncertainties surrounding folate bioavailability, particularly with the use of supplemental folic acid, leaves little confidence that these values are a true representation of the ANR. Another flaw of these studies is the inadequate duration to observe a plateau in erythrocyte folate response. The study of O’Keefe *et al.* [96] was conducted over 10 weeks (70 days) with subjects consuming a consistent diet, while the 13-week (91 day) study of Sauberlich *et al.* [95] involved a 4-week depletion diet followed by three 3-week repletion intervals whereby participants were provided with additional amounts of food folates along with graded doses of supplemental folic acid. Recent efforts to assess long-term blood folate responses to supplemental folic acid have shown that even with a relatively small daily intake of folic acid (140 µg/day) over a 40-week period, erythrocyte folate concentrations continued to increase [99]. Thus, the studies described above may have underestimated the biochemical response to controlled folate intakes and potentially overestimated folate requirements.

Interestingly, nutritional epidemiological evidence consistently demonstrates population intakes that are inconsistent with rates of folate deficiency. For example, most observational population studies have reported average dietary folate intakes ranging between 200 and 300 µg/day (Table 3); yet in a comprehensive review, Metz [100] found little evidence of anemia attributable to folate and vitamin B12 worldwide, with many studies showing no association between blood folate concentration and anemia even when the prevalence of low blood folate concentration is such that a significant association would be expected. It is conceivable that a discrepancy in the prevalence of low folate intakes, low folate status, and megaloblastic anemia exists due to measurement errors in dietary intake and laboratory assessment of folate status as well as a potential overestimation of folate requirements as previously noted.

To date, despite compelling evidence to support recommendations for women who are capable of becoming pregnant or who are planning a pregnancy to consume 400 µg of supplement folic acid/day for the prevention of NTDs, NIVs for women of reproductive potential in most countries are based on prevention of hematological abnormalities. A number of countries have established recommendations in addition their NIVs for the prevention of NTDs, recommending an additional 400 µg/day of folic acid from either supplements or a combination of supplements or fortified foods [16,34,50,52,86,92,93]. One exception is the Nordic countries, while still recommending an additional intake of 400 µg/day from supplemental folic acid [92,93], the Nordic countries have specifically set an INL_98_ of 400 µg/day for women of reproductive potential to reduce their chance of having an NTD-affected pregnancy *versus* the Nordic INL_98_ of 300 µg/day for adults [26]. Establishing an ANR for NPNL women on the basis of NTD prevention remains challenging, as there are a number of uncertainties surrounding the relationship among NTD risk, folate intake (natural food folate and folic acid), and erythrocyte folate concentration. The IOM considered data from two retrospective observational studies [101,102], showing a decrease in the risk of NTDs with increasing intake of dietary folate up to 400 µg DFE/day [52]. There were a number of limitations in these studies including relatively small participant sample size. At present, the minimum intake of folic acid to increase erythrocyte folate to concentrations associated with the lowest risk of NTDs is unknown [8,103].

**Table 3:** Mean and median intakes of folate for women from population survey data by country.

Country	Year	Method	Group	*N*	Mean	Median	Units	Reference.
Australia	1995	24 h RC	19–24 year	575	233	217	µg	[104]
			25–44 year	2385	227	210	µg	
Canada	2004	24 h RC	19–30 year	1854	415 ^1^	401 ^1^	µg DFE	[105]
			31–50 year	2686	423 ^1^	411 ^1^	µg DFE	
Denmark	1995	7 day DR	19–24 year	100	244	220	µg	[106]
			25–34 year	161	266	232	µg	
			35–44 year	158	234	239	µg	
Finland	1997	24 h RC	25–74 year	325	209 ^2,3^		µg	[107]
	2007	48 h RC	25–34 year	180	216 ^3^		µg	[108]
			35–44 year	211	217 ^3^		µg	
		48 h RC & 3 day EDR	25–74 year	641	240 ^3,4^	230 ^3,4^	µg	
					310 ^2,3,4^	280 ^2,3,4^	µg	
Germany	1997–1999	DH	18–79 year	2267		229	µg	[109]
						238 ^2^	µg	
	2005–2006	DH	19–24 year	510	318	257	µg DFE	[110]
			25–34 year	972	311	258	µg DFE	
			35–50 year	2694	285	255	µg DFE	
Ireland	1997–1999	7 day DR	18–35 year	269	247 ^2^	216 ^2^	µg	[111]
			36–50 year	286	267 ^2^	228 ^2^	µg	
New Zealand	1997	24 h RC	19–24 year	209	202	195	µg	[112]
			25–44 year	1205	220	213	µg	
Spain	1992–1993	24 h RC & FFQ	18–34 year	431	282	257	µg	[113]
			35–49 year	323	317	291	µg	
Sweden	1997–1998	7 day EDR	25–34 year	132	209	211	µg	[114]
			35–44 year	132	206	201	µg	
The Netherlands	1992	2 day DR	19–21 year	107	243	236	µg	[115]
			22–49 year	1493	234	224	µg	
	1993–1996	FFQ	20–65 year	1160	192		µg	[116]
	2003	24 h RC	19–30 year	398	153		µg	[117]
	2007–2010	24 h RC	19–30 year	347		216	µg DFE	[118]
						249 ^2^	µg DFE	
			31–50 year	351		242	µg DFE	
						282 ^2^	µg DFE	
United Kingdom	1986–1987	7 day WDR	16–24 year	189	198	194	µg	[119]
			25–34 year	253	206	198	µg	
			35–49 year	385	220	212	µg	
	2000–2001	7 day WDR	19–24 year	104	229	225	µg	[120]
					248 ^2^	232 ^2^	µg	
			25–34 year	210	233	229	µg	
					249 ^2^	233 ^2^	µg	
			35–49 year	318	255	251	µg	
					280 ^2^	258 ^2^	µg	
	2008–2010	4 day EDR	19–64 year	461	232	219	µg	[121]
					264 ^2^	234 ^2^	µg	
United States	1988–1994	24 h RC	20–39 year	2260	217	178	µg	[122]
	1999–2000	24 h RC	20–39 year	356	294	270	µg	[122]
	2003–2006	24 h RC	19–30 year	914	460 ^1^		µg DFE	[123]
					645 ^1,2^		µg DFE	
			31–50 year	1350	470 ^1^		µg DFE	
					714 ^1,2^		µg DFE	

DFE: dietary folate equivalent; DH: diet history; DR: diet record; EDR: estimated diet record; FFQ: food frequency questionnaire; RC: recall; WDR: weighed diet record; ^1^ intake is measured following mandatory fortification of the food supply with folic acid; ^2^ inclusive of intake from supplements; ^3^ adjusted for folate losses in cooking; ^4^ excluding under-reporters.

Observational studies of dietary intake are subject to systematic errors that often lead to an underestimation of usual folate intakes due to underreporting of food intake and inaccuracies in food composition databases, secondary to methodological issues in analysis [52,124,125]. Furthermore folic acid intake from supplements and fortified foods, an important contributor to biochemical folate status, is not always considered in reports of dietary intake [125]. Thus determination of NIVs based on metabolic studies of controlled folate intake may result in higher values than those based on observational studies of dietary intake. The U.S. RDA for women for folate was lowered from 400 µg/day in the ninth edition (1980) to 180 µg/day in the tenth edition (1989) based on the recognition that observed population intakes of roughly 3 µg/kg maintained adequate folate status in approximately 90% of the population [87]. In 1998 the U.S. RDA was increased once again to 400 µg DFE/day based on data from controlled intake studies [52]. A number of countries have used observational data from population studies to set requirements including the UK, Belgium, France, and the Nordic countries.

The disparity between requirements based on studies of controlled folate intake *versus* observational survey data is prevalent and can be a significant hindrance to the way science informs and influences public health policymaking (e.g., food fortification programs). The ongoing development and validation of innovative dietary assessment methods (e.g., internet-based assessment, and the use of digital cameras and cellular phones), and improvements in analytical measurement of food and tissue folate levels will provide more accurate and meaningful data. Until then, caution is warranted in interpreting intake-status results from epidemiological studies.

### 4.2. Pregnant Women

Because of ethical considerations, depletion-repletion studies in pregnant women are lacking. Consequently, folate requirements for pregnant women are largely based on findings of population-based supplementation trials conducted in the 1960s [4,126,127,128] and a more recent controlled metabolic study aimed to evaluate the adequacy of the current folate requirements in a group of pregnant women [129]. Estimates generated from these earlier supplementation trials determined that the minimum daily requirement of supplemental folic acid in pregnant women was approximately 100 µg/day [4,126]; however, if megaloblastic anemia was more common in the population (*i.e.*, in a population with suboptimal folate intake), a daily supplement of 300 µg folic acid/day was closer to the minimum requirement, particularly in late pregnancy [7,127,128]. Specifically, Hansen and Rybo [4] demonstrated that while supplementation with 50 µg folic acid/day taken in the last trimester of pregnancy was not sufficient to maintain maternal folate stores, 100 µg folic acid/day was found to be adequate to prevent against a decline in erythrocyte folate concentration, however 15% of study participants had serum folate levels below 2 ng/mL. Willoughby and Jewel [128] found that 100 µg folic acid/day was inadequate to prevent a serum folate concentration below 3 ng/mL in 33% of women compared to only 5% of women supplemented with 300 µg folic acid/day. Furthermore, none of the participants consuming 300 µg folic acid/day presented with megaloblastic anemia compared to 2% of the group supplemented with 100 µg folic acid/d. In a larger study of 3599 pregnant women, Willoughby [127] found that supplementation with 300 µg folic acid/d reduced the rate of megaloblastic anemia to 0.3% compared with 0.7% reported in a similar study of women (*n* = 350) supplemented with 100 µg/day. In contrast, Chanarin *et al*. [126] showed that supplementation with 100 µg folic acid/day was sufficient to maintain both serum and erythrocyte folate concentration during the last trimester of pregnancy in a group of 206 British women. The discrepancies in these findings may be explained by differences in dietary folate intakes. However, the dietary intakes of study participants were not assessed, with the exception of Chanarin *et al*., who determined an average baseline dietary folate intake of 676 µg of total folate/day [130]. This unexpectedly high value was derived from 24-h food collection obtained from a subsample of participants (16 of 206 women). Given that these studies were conducted prior to folic acid fortification of the food supply, usual dietary folate intakes were likely to be lower, ranging from 200 to 300 µg/day [131].

Caudill *et al*. conducted the only controlled metabolic study of folate intake [129]. Pregnant participants (*n* = 12) in their second trimester and non-pregnant controls were randomly assigned to supplemental folic acid intakes of either 330 or 730 µg/day, in addition to a diet containing 120 µg/day of dietary folate, for 12-weeks (84 days). No differences were detected in blood folate status between pregnant women and non-pregnant controls within the same supplementation group. Moreover, no women presented with a low serum (<13.6 nmol/L) or erythrocyte (<364 nmol/L) folate throughout the duration of the study. The authors concluded that 450 µg/day (dietary folate plus supplemental folic acid), equivalent to 600 µg DFE/day, was sufficient to maintain folate status in pregnant women. Using the equation for DFEs proposed by the IOM, this value is actually equivalent to 680 µg DFE/day. From these data along with the findings of the population studies, the IOM derived their ANR by adding 200 µg DFE to the ANR for NPNL women (320 µg/day DFE) to provide an ANR for pregnant women of 520 µg DFE/day. Assuming a CV of 10% and based on the support of the controlled metabolic study, the INL_98_ was set at 600 µg DFE/day [52]. Similarly, a number of other countries including the European Community, the DACH countries, and the Netherlands, have established NIVs based primarily on data from the population-based supplement trials with an addition of 200 µg folate added to their NIVs for NPNL women [28,50,86]; however differences in NIVs for pregnant women among these countries ranging from 400 to 600 µg/day have arisen directly from differences in NIVs set for NPNL women.

In contrast, several Nordic countries (Denmark, Sweden, and Iceland) have established a slightly lower recommendation of 500 µg/day [26] on the basis that requirements would not be as high as the minimum dose of 600 µg DFE/day in the study of Caudill *et al*. [129], particularly given the observed increase in erythrocyte folate status. India has also set their INL_98_ of 500 µg DFE/day although this value was based on the country-specific findings of a supplementation trial in 200 Indian pregnant women assigned to 60 mg of elemental iron with or without folic acid at levels of 100, 200, or 300 µg/day [132]. While a daily intake of 100 µg folic acid was sufficient to maintain erythrocyte folate concentrations, mean birth-weight was significantly higher in the 300 µg folic acid/day group *versus* the iron only group. A cross-sectional study of a group of unsupplemented women presented in the same article reported 23% and 64% of women had a serum folate below 3 ng/mL in the first and third trimester of pregnancy, respectively. Based on these findings, it was recommended that pregnant women would require an additional 300 µg DFE/day—although despite the use of DFE units, it does not appear that an adjustment for the bioavailability of supplemental folic acid was made. Finally, Finland and Norway adapted one of the lowest recommendations of 400 µg/day, with the reasoning that folate deficiency is rare and adoption of a higher level (as specified in the Nordic Nutrition Recommendations) would require the use of folic acid supplements during pregnancy and lactation [29].

Identification of alternative biomarkers of normal folate status that are sensitive to change and specific for folate inadequacy would be highly valuable in contributing to our understanding of the physiologic changes of pregnancy and the influence of dietary folate intake. Some researchers have proposed that the rate of urinary folate catabolite excretion reflects the role of folate in DNA biosynthesis and cellular turnover; therefore, urinary folate catabolites have been suggested as a potential indicator of folate requirements [133]. Specifically, McPartlin *et al*. [133] reported significantly greater urinary excretion of folate catabolites in the second and third trimester than in the first trimester, post-partum or NPNL state. Using a 50% bioavailability factor for dietary folate, the authors calculated an extra demand for dietary folate of about 200–300 µg/day during pregnancy. In contrast, the controlled metabolic unit study conducted by Caudill *et al*. [129,134], found no significant differences in mean folate catabolite excretion between pregnant women in the second trimester and non-pregnant controls; however catabolite excretion was significantly higher in pregnant and non-pregnant women consuming 850 *versus* 450 µg folate/day [134]. It was also noted that the pregnant women consuming 450 µg folate/day experienced a decline in urinary catabolite excretion from baseline compared to their non-pregnant counterparts. This decline in urinary folate catabolite excretion may reflect a decrease in supplemental folate intake rather than folate requirement. Prior to the study, 10 out of 12 pregnant women were consuming folic acid supplements between 400 and 1000 µg/day [129]. Whereas in the study of McPartlin *et al*. folate intake was only controlled up to 18 h before the collection of the 24-h urine sample and folic acid supplement use was not reported [133]. Current evidence cannot rule out whether the differences observed between these two studies are due to the different gestational periods of the women or are a reflection of prior supplemental intake. Additional work from metabolic controlled studies measuring changes in urinary folate catabolite excretion throughout pregnancy under conditions of inadequate folate intake to optimal intake levels are needed.

Other health outcomes related to maternal folate intake and status during pregnancy have been examined, with several studies finding a relation with birth weight and gestational age [135,136,137,138,139,140] whereas other have not [141]. In a recent meta-analysis, increased folate intake was found to be significantly associated with birth weight in a dose-dependent manner, but had no effect on placental weight or length of gestation [142]. Although data related to such perinatal outcomes are of potential interest, the inconsistent findings demonstrate the multi-factorial nature of these outcomes.

### 4.3. Lactating Women

Unlike NIVs for NPNL and pregnant women that are based on maintenance of blood folate status, NIVs for lactating women are often calculated as the sum of folate requirements of NPNL women and the amount of folate lost in breast-milk, with adjustments made for bioavailability. In doing so, variation in NIVs among countries can be partly attributed to differences in NIVs for NPNL women, and partly to differences in bioavailability adjustment factors and estimations of human milk folate content and volume [143]. Finland, Norway and France have not set NIVs specifically for lactating women, and have assigned NIVs using the same rationale as for pregnant women.

Table 4 shows the data used and calculations made by a number of countries to determine folate NIVs for lactating women based on extrapolation of human milk folate concentrations. Due to analytical difficulties, early measurements of human milk folate content were underestimated [52]. The U.K. based its NIV for lactating women on the study of Ek [144] published in 1983 which demonstrated a steady rise in milk folate concentration from 6 µg/L at 0 months after parturition up to 55 µg/L at 3 months after parturition; however, higher average milk folate values (average of 85 µg/L) were reported by Smith *et al*. in 1985 [145]. Later studies published include those of: Brown *et al*. [146], which reported a mean concentration of 85.3 µg/L in 180 samples from 16 women; O’Connor *et al*. [147], which reported slightly higher values between 90 and 110 µg/L in samples from four women after optimizing sample pre-treatment; and Lim *et al*. [148], which reported a mean concentration of 86 µg/L in 84 human milk samples. These three studies are cited in the reports of the IOM and the Netherlands although it is unclear why the Netherlands used a lower milk folate concentration of 60 µg/L. Human milk folate concentrations ranging between 70 and 110 µg/L have been confirmed in more recent studies using appropriate sampling techniques and pretreatment protocols [149,150,151,152].

**Table 4:** Data used to derive nutrient intake values (NIVs) for lactating women from breast-milk losses of folate by country ^1^.

Country	Milk Folate Concentration	Daily Milk Production	Losses Per Day	Adjustment for Bioavailability	Addition in Requirement ^2^	Added to ANR or INL_98_
Austria, Germany and Switzerland ^3^	80 µg/L	750 mL/day	60 µg/day	50%	120 µg DFE/day	ANR
European Community	50–100 µg/L	750 mL/day	37–75 µg/day	50%	150 µg/day	INL_98_
The Netherlands	60 µg/L	800 mL/day	48 µg/day	50%	100 µg DFE/day	INL_98_
Denmark, Iceland and Sweden	60–85 µg/L	750 mL/day	nc	50%	100 µg/day	INL_98_
United Kingdom	−	−	40 µg/day	66%	60 µg/day	INL_98_
United States and Canada	85 µg/day	780 mL/day	66 µg/day	50%	130 µg DFE/day	ANR

ANR: average nutrient requirement; DFE: dietary folate equivalents; INL: individual nutrient level; nc: not calculated in report; ^1^ NIVs are calculated as an additional requirement that is added to the NIV for NPNL women; ^2^ values may be rounded; ^3^ proposed values to be approved August 2013 [86].

The factorial approach used to determine NIVs for lactating women assumes that milk folate content can be used as a proxy for maternal folate adequacy. However, evidence indicates that adequate milk folate content is maintained during folate depletion states [5]. While there is some evidence to suggest that blood folate status of lactating women decreases as lactation progresses [153,154], further longitudinal studies are needed to confirm this observation. It has been suggested that folate status of the lactating mother in these study populations often declines merely due to the discontinuation of prenatal folic acid supplements [154]. A number of studies have been carried out on maintenance of blood folate status during lactation [76,152,155,156,157]; however, these studies have also been conducted in groups of women who had consumed high-dose folic acid supplements throughout pregnancy.

Lastly, NIVs for lactation do not account for the influence of birth spacing on maternal nutritional status. Short intervals between pregnancies have been associated with increased risk of preterm birth, low birth-weight, and small-for-gestational-age babies [158]. Although the cause of these poor reproductive outcomes among women with short birth spacing has been debated, one plausible hypothesis is that women with closely spaced births have insufficient time to restore the folate reserves needed to support optimal fetal growth and development in the subsequent pregnancy [159]. Folate supplementation during early and late pregnancy has been shown to reduce the association between short inter-pregnancy spacing and low-birth weight [160]. In populations where supplementation rates during pregnancy are low, it may be of benefit to achieve an intake level that maintains blood folate status during both pregnancy and lactation.

## 5. Conclusions

NIVs set by different countries for folate vary substantially. Among countries, the range of INL_98_ levels were 160–460 µg/day for NPNL women, 300–750 µg/day for pregnant women, and 260–650 µg/day for lactating women. Differences in the indicators selected, the criteria for adequacy, and the type of evidence and assumptions made to set recommendations are all likely to contribute to the variation; however, the lack of transparency in the decisions made ultimately hampers the understanding of the differences [161]. The need to standardize nutrient requirements has also been recognized by the EURopean micronutrient RECommendations Aligned (EURRECA) Network of Excellence in 2007. Although funding for EURRECA ended in 2012, the primary aim of the initiative was to provide a harmonized approach to guide the process of deriving micronutrient requirements in the European populations. Activities included the development and dissemination of a framework outlining the ideal process for deriving dietary reference values in a transparent, systematic and scientific way [162]. Despite the WHO/FAO recommendations on establishing a common terminology for NIVs, it was felt for practical purposes that establishing a common European terminology may lead to miscommunication and misinterpretation of NIVs at a national level [162]. EURRECA have also established a list of priority nutrients for review on the basis of: the amount of new evidence available; public health relevance of the nutrient; and variation in current recommendations of different European countries [163]. Folate was ranked in the top ten nutrients prioritized, fulfilling all three criteria in all life-stage groups, except for infancy, for which folate did not meet the criteria of public health relevance. However, despite an ongoing accumulation of evidence linking folate status with health outcomes, undertaking a formal review of folate requirements may prove difficult. A recent systematic review performed to support revision of the NNR folate requirement concluded that there was insufficient quality evidence to question current folate recommendations [164]; regardless, revision of older NIVs, such as those set by the UK (1991), European Union (1993), and the IOM (1998), may be justified. There is a lack of evidence for differences in requirements of folate among countries for NPNL women, and pregnant and lactating women in well-nourished populations; therefore achieving consensus in NIVs supported by a wide evidence base should be a priority.

Although challenging, additional studies in these life-stage populations groups are urgently needed. Most of the evidence supporting folate NIVs has been limited by data from relatively small sample sizes and uncertainties in bioavailability factors. Issues regarding laboratory measurement of food folate and biochemical folate status still exist and will need to be resolved in order to accurately determine dietary intake and provide definitive conclusions on the extent of folate deficiency. Once these issues are resolved, further studies are needed to evaluate the metabolic consequences associated with inadequate folate intakes and the reversal of these changes with increased intake levels. Controlled-metabolic studies are not feasible in large samples nor for a period of time long enough to observe a plateau in blood folate response; however it is possible to set NIVs based on observational data if intake data are accurate and captured over a sufficient period of time to ensure the sample days are truly representative of usual intake. For NPNL women, long-term observational studies of dietary folate intake in relation to erythrocyte concentration should not only be adequately powered to provide a precise estimate of the average requirement, but also to determine the variation in requirements. Given the importance of folate in prevention of NTDs during preconception, a higher erythrocyte folate status may serve as a better indicator of adequacy in NPNL women than erythrocyte folate status required to prevent megaloblastic anemia.

For pregnant women, there is reasonably good evidence that an additional 100 µg folic acid/day above the requirement for NPNL women is adequate to prevent against a decline in blood folate in a population with adequate intakes and a low prevalence of megaloblastic anemia. Differences among countries NIVs for pregnant women are largely due to differences in NIVs of NPNL women. Studies of folate requirements in pregnant women designed to maintain optimal folate status will be confounded by the use of high-dose supplements in the range of 400–1000 µg folic acid during preconception and early pregnancy [76,129,152,165]. It is important to determine future requirements based on an optimal erythrocyte folate level rather than maintenance of status in such populations, and to consider the effect of high-dose supplements on subsequent folate requirements throughout pregnancy. Similarly, this confounding effect should also be considered when evaluating folate status data during lactation. The current approach for deriving folate requirements during lactation with use of breastmilk folate content as a proxy for maternal adequacy may be inappropriate. Further research designed to evaluate the relationship between folate intake and blood folate status or other indicators during lactation among previously un-supplemented women are required.
